# Reduction of seed motion using a bio‐absorbable polymer coating during permanent prostate brachytherapy using a mick applicator technique

**DOI:** 10.1002/acm2.12254

**Published:** 2018-04-17

**Authors:** Gregory R. Warrell, Yan Xing, Tarun K. Podder, Bryan J. Traughber, Rodney J. Ellis

**Affiliations:** ^1^ Department of Radiation Oncology University Hospitals Cleveland Medical Center Cleveland OH USA; ^2^ Case Western Reserve University Cleveland OH USA

**Keywords:** brachytherapy, prostate seeds, vicryl, seed motion, seed migration, seed slippage, seed fixity

## Abstract

**Purpose:**

The addition of a braided bio‐absorbable vicryl coating to the surface of radioactive seeds used for low dose rate (LDR) prostate brachytherapy is intended to reduce the incidence of seed movement and migration. Here, we present a single‐institution study of the frequency and severity of seed slippage (initial seed movement) of coated seeds in comparison with uncoated seeds.

**Methods:**

Forty‐seven patients received permanent prostate brachytherapy, with either coated (*n *=* *26) or uncoated (*n *= 21) seeds. AgX100 ^125^I seeds, coated or uncoated, and uncoated Model 200 ^103^Pd seeds were used. During the ultrasound‐guided implantation procedure, each implanted seed was categorized as having remained in the implanted position after being placed, having moved slightly, or having left the ultrasound field of view.

**Results:**

3.1% of the coated seeds (AgX100 seeds, *n *= 70) and 6.9% of the uncoated seeds (AgX100 and Model 200 seeds, *n *= 128) were observed to have moved at least 2 mm from their initial implant positions, respectively. The difference in incidence of this movement was 54.4% (*P* = 0.0026). Coated AgX100 seeds demonstrated a 66.7% lower rate of movement of at least 2 mm than that for uncoated AgX100 seeds (*P* = 0.038), and a 49.0% lower rate than that for Model 200 seeds (*P* = 0.021). While no significant differences were noted in prescription dose coverage of the prostate or the studied dosimetric parameters for the organs at risk between the coated and uncoated seeds (*P* > 0.05) in the CT‐based Day‐0 postoperative plans, the limited sample size and differences in energies between the ^125^I and ^103^Pd seeds make further analysis of postoperative dosimetric coverage difficult without additional data directly comparing the coated and uncoated ^125^I seeds.

**Conclusion:**

When the vicryl coating is used, seeds have a significantly lower propensity to slip from their initial implant locations. This may help maintain dosimetric integrity, warranting further study of postoperative dosimetry.

## 1 INTRODUCTION

Permanent prostate brachytherapy (PPB) is a highly effective modality for patients with localized prostate cancer, with favorable treatment outcomes and biochemical control in comparison with external beam radiation therapy (EBRT).^1^ Consequently, PPB has become a standard treatment for treating early‐stage prostate cancer and as a boost in men with high‐risk disease.^2^, ^3^, ^4^

Slippage or initial seed movement from the intended point of implant, however, is a well‐known phenomenon with PPB.^5^ Placement of seeds into unintended positions may negatively affect real‐time and postimplant dosimetry, and suboptimal seed fixity may increase the incidence of seeds dislodging into adjacent blood vessels,^6^ where they may be transported by blood flow to distant anatomical sites, such as the lungs, heart, and other organs. It is, therefore, desirable to minimize seed slippage and movement in the PPB procedure.^7^, ^8^, ^9^, ^10^

One way of improving seed fixity is to use linked or stranded seeds. It has been reported that the use of the stranded seeds, in which the seeds are encapsulated within a bio‐absorbable strand of flexible polymer, leads to reduced chances of seed migration.^11^ However, stranded and linked seeds are not free of limitations. Intra‐operative linkage of seeds may lengthen the operation time. In addition, the lack of flexibility of a strand of uniformly spaced seeds limits the capability of customized design of seed pattern toward regions of suspected tumors and away from critical structures. There are also concerns that postimplantation edema of the prostate followed by edema resolution may cause implanted strands to collapse, preventing them from covering the whole length of the gland.^12^ Furthermore, the positions of seeds within pre‐ordered fixed‐spaced strands cannot be modified once the strands are created, meaning that if there is a substantial change in the shape of the target or organs at risk between that of the pre‐operative plan, and as determined after inter‐operative image acquisition, the ability to modify the plan accordingly is limited. While implants combining stranded and free seeds have been performed,^13^ there remain dosimetric advantages in the exclusive use of free or loose seeds in prostate brachytherapy.

An alternate means of improving seed fixity is through the use of a bio‐absorbable polymer encapsulation of each seed, which is intended to prevent the smooth titanium outer shell of the seed from moving along and through soft tissue. The coating is sufficiently thin to permit the seeds to be used in a standard Mick^®^ applicator and needle (Mick Radio‐Nuclear Instruments, Inc., Bronx, NY, USA). It has previously been shown that such a coating improves the fixity of seeds in that it reduces their movement within the target region, preserving target coverage and organ at risk (OAR) sparing.^7^ The present study quantifies the incidence of local initial movement (slippage) of seeds as visualized in real‐time ultrasound imaging with a proprietary braided coating in comparison with uncoated seeds with a Mick^®^ applicator technique.

## 2 METHODS

### 2.A The mick applicator system and polymer seed coating

For this institutional study, the IRB approved a retrospective review of the medical records of all study subjects diagnosed with localized prostate carcinoma (T1c‐2bNxM0) who underwent transrectal ultrasound (TRUS) brachytherapy with a Mick applicator as performed by radiation oncologists in our clinic using a previously described technique.^6^, ^14^ In short, either Theragenics (Theragenics Corp., Buford, GA, USA) model AgX100 ^125^I seeds or Model 200 “Theraseed^®^” ^103^Pd seeds were placed freely with a Mick applicator under TRUS guidance. If ^125^I seeds were used, the prescribed dose was either 145 or 110 Gy, the former if done as a monotherapy and the latter if done as a boost to external beam radiation therapy (EBRT). The corresponding prescribed doses for ^103^Pd seeds were 125 and 100 Gy, respectively.

**Figure 1:**
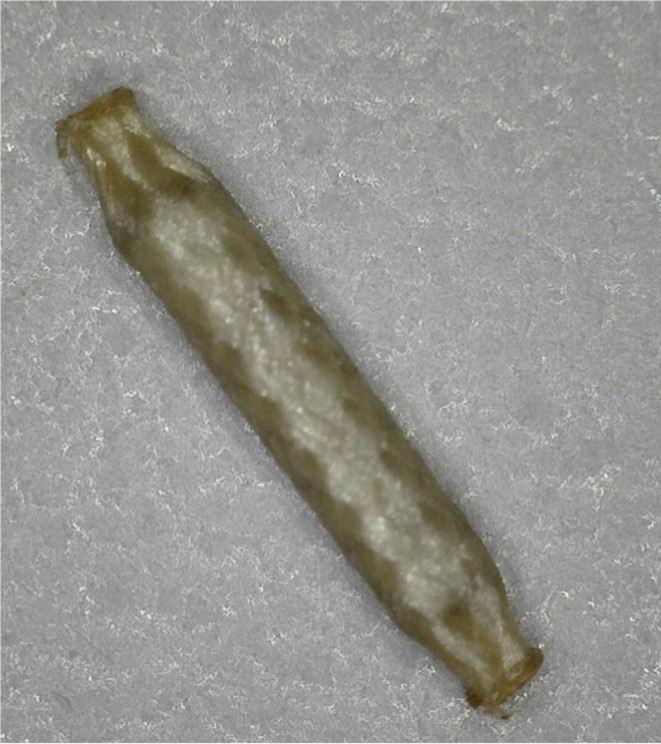
Braided vicryl seed encapsulation used with AgX100 seed as used in this study. The nominal seed length before encapsulation is 4.5 mm.

The air kerma strength per seed at the time of implant was typically ~0.5 U for ^125^I seeds and ~1.8 or ~2.3 U for ^103^Pd seeds, the former if the implant with ^103^Pd seeds was done as a boost and the latter if done as a monotherapy. Occasionally, if the number of needles called for by the pre‐operative plan was particularly high (>30), a higher seed activity was used to reduce the number of needles required for PPB, lessening needle‐related trauma. In most of the cases that the prescribed isotope was ^125^I, seeds with a polymer coating were used; this was the Theragenics TheraStrand Single Load (TSL) 90/10 glycolide/L‐lactide (vicryl) braided seed encapsulation^15^ (Fig. 1). The nominal coating thickness (before absorbing water and expanding within tissue) is 0.0825 mm, with a density of 1.507 g/cc.^15^ When AgX100 seeds were used for an implant, either all coated or all uncoated seeds were used; there was no mixing of coated and uncoated seeds in the same implant.

All treatment planning was done in the treatment planning software package MIM Symphony^TM^ version 6.3.9 (MIM Software Inc., Cleveland, OH, USA), using published line‐source TG‐43 values for the AgX100 and Model 200 seeds.^16^, ^17^ The TG‐43 values published for the uncoated AgX100 seeds were used for the coated seeds, as the manufacturer had assured our group that an internal Monte Carlo study had shown no discernable difference in the TG‐43 values for the cases of a coated and uncoated seed. Moreover, another group using ^125^I seeds with a coating of similar composition and thickness from another manufacturer experimentally found an attenuation factor of the coating of 0.2%, demonstrating its negligible effect on dosimetry.^18^

Several dosimetric plans were generated for each patient at various stages of the planning and implantation process. After acquisition of images for a volume study (typically T2 MRI images), a preprocedural plan (pre‐operative plan) was generated, which was used to determine the appropriate number of seeds and seed activity to order. In the operating room, after the patient had been anesthetized and ultrasound (US) images acquired with the TRUS probe, the pre‐operative MR images and plan were co‐registered to the US images and the plan modified (if needed) to create an intra‐operative plan. The seed implantation was monitored in real time and the plan dosimetry was updated during the implant, adjusting the positions of the needles and the seeds as they were implanted to match their positions as visualized in real‐time US images, resulting in a real‐time plan.^19^ This was to provide real‐time clinical evaluation of the implant achieved thus far, informing any decisions made to implant additional seeds prior to ending the case and awakening the patient. Finally, a postprocedural plan was generated based on seed positions visualized in the same‐day CT images taken after patient recovery. In order to assure the integrity of images used in the clinic, periodic quality assurance was performed on the US unit and CT scanner according to the protocols promulgated in the AAPM task group reports TG‐128 and TG‐66, respectively.^20^, ^21^

Both intra‐operative modification of preprocedural dosimetry and intra‐operative dose escalation were performed in regions felt to be at high risk for occult tumor localization based on pre‐operative multiparametric MRIs (mpMRIs).^22^ In such areas, up to 150% of the prescribed dose was delivered to regions of clinical suspicion based on pre‐operative mpMRIs.

Based on the results of the dynamically adjusted real‐time plan (particularly D90 and V100 to the prostate contour), fluoroscopic images, and overall coverage of the prostate by seeds as visualized in the TRUS images, a clinical decision was sometimes made to add one or more additional seeds to regions felt to be at risk for under‐coverage. Fluoroscopic images were also used and cytoscopy was performed to recover any seeds inadvertently placed in the bladder wall. However, this was not routinely required as the open needle used during placement of seeds with the Mick applicator permitted urine to pass from the needle if the tip were positioned in the bladder; any such needle was retracted prior to source placement.

During seed placement, ratings were concurrently provided by the radiation oncologist based on the real‐time sagittal plane ultrasound imaging. If, according to the judgment of the treating radiation oncologist with the agreement of the medical physicist, a seed initially stayed within 2 mm of where it had been originally placed upon exiting the Mick applicator needle (i.e., minimal slippage), a grade “A” was given. A seed was given a “B” rating if it was visualized to have deviated by more than 2 mm from the initial position of implantation, up to about 5 mm. If a seed had moved more than 5 mm or sufficiently to take it out of the field of view of the sagittal plane ultrasound image (considered to be at least 5 mm), a “C” grade was given. In order to eliminate inter‐observer variation, the same observer provided ratings of seeds as they were placed. All observations and grades of seed slippage were made on the ultrasound images only.

TRUS images used to perform the implant and guide the slippage ratings were acquired with a BK Medical Flex Focus 400 ultrasound used with a Type 8848 endocavity biplane transducer (BK Medical Systems, Inc., Peabody, MA, USA). The manufacturer reports that at the 12 MHz B‐mode frequency used in our clinic for PPB, this combination provides sagittal‐plane images with axial (anterior–posterior direction) and lateral (superior–inferior direction) spatial resolutions of 0.5 and 1.0 mm, respectively.^23^ These images were obtained with the transducer's 192‐element sagittal linear array, which has a 5.5‐mm‐wide aperture and 65 mm length. In accordance with the periodic quality control tests described in the AAPM task group report TG‐128, annual measurements of axial and lateral resolution had been obtained by imaging small high‐contrast targets within a quality assurance phantom. No discernable change in resolution over that reported by the manufacturer had been observed, a result more than satisfying the <1 mm control limit in measured change in spatial resolution recommended by TG‐128.^20^ To further assure the accuracy of images acquired during the implant procedure, preprocedural daily quality assurance was conducted of the functionality of the stepper encoder, agreement of the digital templates in the ultrasound display and planning software, and of the clinical acceptability of the US images once the transducer was inserted.

After postoperative recovery on the day of seed implantation (Day‐0), all patients received CT scans of the pelvis. Seed placement and any potential migration were assessed and implant dosimetry performed. Prior to discharge, postprocedure surveys were conducted of the voided urine to detect any seeds that had been passed through the urethra.

### 2.B Study population and data collection

Retrospective analysis was traced back to the first patient who had received PPB with the polymer coating in our clinic on January 6, 2016. Data were collected for 47 patients who received brachytherapy seed implants with or without the polymer coating between November 11, 2015, and November 30, 2016. The same radiation oncologist and medical physicist, each with 15–20 yr of current experience in our high‐volume institution, conducted all implant procedures studied here.

Patient characteristics of the group receiving coated seeds and that receiving uncoated seeds were constructed and compared. The number of seeds that had received “B” or “C” ratings as visualized on the real‐time ultrasound imaging was recorded and compared between the two groups. On the postoperative CT images, the dosimetric profiles of the prostate volumes as well as organs at risk of the coated and uncoated groups were also analyzed and contrasted, including key dosimetric indicators such as V100, V150, V200, and D90 of the prostate; V100, V150, and D10 of the prostatic urethra; and D0.1 cc, D1 cc, and D2 cc of the rectum.

### 2.C Statistical analysis

The patient characteristics, postoperative dosimetric profiles, and the incidences and degree of seed slippage were compared between the coated and uncoated groups using the two‐tailed heteroscedastic Student's *t*‐test. A *P*‐value less than 0.05 was considered statistically significant. Similar comparisons were also made between coated and uncoated AgX100 seeds only as well as between coated AgX100 seeds and uncoated Model 200 seeds.

## 3 RESULTS

### 3.A Patient characteristics

Twenty‐six patients who were implanted with coated seeds all received ^125^I seeds, while of the 21 who received uncoated seeds, 4 received ^125^I seeds and 17 received ^103^Pd seeds. The number of implantation needles used in each case ranged from 17 to 35 (median of 26), with 62 to 127 seeds implanted per patient (median of 83). Most patients had low‐ to intermediate‐risk prostate cancer; among the studied patients, the Gleason scores varied between 6 and 9, the preprocedural prostate‐specific antigen level between 3.6 and 64.82 ng/mL, the primary tumor stage between T1c and T3a, and the anatomic stage/prognostic Group between I and III.

Twenty‐six implants using coated seeds, all ^125^I, were conducted between January 6, 2016, and November 30, 2016, while the 21 using uncoated seeds, whether ^103^Pd or ^125^I, were performed between November 11, 2015, and November 9, 2016. The four implants with uncoated ^125^I seeds were all performed in 2015, while the 17 with uncoated ^103^Pd seeds were performed in 2015 and 2016.

Among the patients implanted with coated AgX100 seeds, two patients had Group I disease and one had Group IIB disease, the rest being in Group IIA. Three of the four patients who received uncoated AgX100 seeds were in Group IIA, with the other in Group I. The patients who received Model 200 seeds were predominantly in Group IIA as well, with two in Group I, three in Group IIB and one in Group III. A more detailed comparison among the studied groups is in Table 1, which finds no significant difference between groups in patient age at the time of implant, PSA level at the time of treatment, the number of seeds placed per patient, or the number of needles used per implant. Some significant differences were found between groups in the proportion of patients receiving treatment as a monomodality and in the volume of the prostate as drawn on the intra‐operative plan.

**Table 1:** Averages, standard deviations, and *P*‐values (where applicable) of characteristics among the patients of this study. *P*‐values below the threshold of significance *P* = 0.05 are highlighted in bold

	Staging of patients at the time of treatment	Patient age at the time of treatment, years (avg ± SD)	PSA at the time of treatment, ng/mL (avg ± SD)	Percentage of patients receiving implant as a monotherapy	Intra‐operative prostate volume, cc (avg ± SD)	Number of seeds placed (avg ± SD)	Number of needles used (avg ± SD)
Coated AgX100 (*n* = 26)	2 at Stage I, 23 at Stage IIA, 1 at Stage IIB	68.0 ± 7.3	7.2 ± 4.0	96.2%	42.3 ± 16.4	86.4 ± 17.0	26.0 ± 3.5
Uncoated seeds, both models (*n* = 21)	3 at Stage I, 14 at Stage IIA, 3 at Stage IIB, 1 at Stage III	67.2 ± 6.7	11.8 ± 13.1	57.1%	36.9 ± 16.5	89.3 ± 19.5	26.1 ± 4.4
Uncoated AgX100 (*n* = 4)	1 at Stage I, 3 at Stage IIA	70.7 ± 11.6	8.4 ± 2.6	100%	55.1 ± 22.2	103.8 ± 24.1	29.0 ± 5.5
Uncoated Model 200 (*n* = 17)	2 at Stage I, 11 at Stage IIA, 3 at Stage IIB, 1 at Stage III	66.4 ± 5.2	12.6 ± 14.5	47.1%	32.6 ± 12.0	85.9 ± 17.3	25.4 ± 4.0
Result of t‐test between coated and all uncoated seeds (*P*‐value)	N/A	0.70	0.14	**0.0020**	0.27	0.60	0.94
Result of t‐test between coated and uncoated AgX100 seeds (*P*‐value)	N/A	0.68	0.47	0.33	0.34	0.25	0.35
Result of t‐test between coated seeds and uncoated Model 200 seeds (*P*‐value)	N/A	0.40	0.15	**0.0013**	**0.031**	0.92	0.61

### 3.B Dosimetric profiles

A comparison of the postoperative dosimetric parameters among the studied groups is shown in Table 2. Among the dosimetric parameters examined, significant differences between groups were found only in V150 and V200 of the prostate. No significant differences were found for D90 to the prostate, V100 to the prostate or prostatic urethra, V150 or D10 to the prostatic urethra, or in D0.1 cc, D1 cc or D2 cc to the rectum.

**Table 2:** Averages and standard deviations of dosimetric parameters for the postoperative CT‐based plans created for all patients, and the results of Student's *t*‐tests for these parameters among the studied groups. P‐values that fall below the threshold of significance *P* = 0.05 are highlighted in bold

	**V100 Prostate**	**V150 Prostate**	**V200 Prostate**	**D90 Prostate**	**V100 Urethra**	**V150 Urethra**	**D10 Urethra**	**D0.1 cc Rectum**	**D1 cc Rectum**	**D2 cc Rectum**
Coated AgX100 (*n* = 26)	94.2% ± 4.0%	48.6% ± 10.5%	20.3% ± 5.0%	109.2% ± 8.3%	82.3% ± 19.7%	9.2% ± 18.0%	141.8% ± 38.8%	91.5% ± 25.1%	65.5% ± 15.8%	55.8% ± 13.2%
Uncoated seeds, both models (*n* = 21)	93.3% ± 4.2%	58.0% ± 9.6%	31.5% ± 8.5%	107.6% ± 9.7%	69.2% ± 24.3%	13.3% ± 16.7%	149.8% ± 35.0%	94.4% ± 43.5%	60.0% ± 25.0%	48.1% ± 20.0%
Uncoated AgX100 (*n* = 4)	94.0% ± 4.0%	47.0% ± 9.3%	21.3% ± 6.1%	106.0% ± 6.5%	70.9% ± 19.9%	11.2% ± 12.1%	151.3% ± 37.1%	90.2% ± 17.1%	66.7% ± 10.8%	57.4% ± 8.3%
Uncoated Model 200 (*n* = 17)	93.2% ± 4.4%	60.6% ± 7.9%	33.9% ± 7.1%	108.0% ± 10.4%	68.8% ± 25.8%	13.7% ± 17.9%	149.4% ± 35.7%	95.3% ± 48.0%	58.4% ± 27.4%	45.9% ± 21.4%
Result of t‐test between coated and all uncoated seeds	*P* = 0.45	***P*** ** = 0.0025**	***P*** ** = 7.1 × 10** ^**−6**^	*P* = 0.55	*P* = 0.053	*P* = 0.43	*P* = 0.46	*P* = 0.79	*P* = 0.39	*P* = 0.13
Result of t‐test between coated and uncoated AgX100 seeds	*P* = 0.91	*P* = 0.77	*P* = 0.76	*P* = 0.42	*P* = 0.35	*P* = 0.78	*P* = 0.66	*P* = 0.90	*P* = 0.86	*P* = 0.77
Result of t‐test between coated seeds and uncoated Model 200 seeds	*P* = 0.42	***P*** ** = 0.00011**	***P*** ** = 2.4 × 10** ^**−7**^	*P* = 0.69	*P* = 0.077	*P* = 0.43	*P* = 0.51	*P* = 0.76	*P* = 0.35	*P* = 0.099

### 3.C Seed slippage incidence

**Figure 2:**
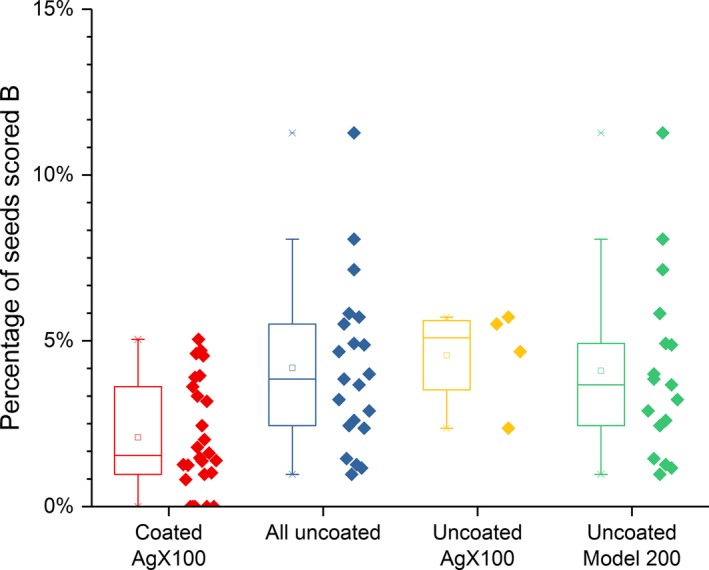
Percentages of seeds scored B for each implant, for implants using coated seeds, uncoated seeds of both nuclides, uncoated AgX100 seeds, and uncoated Model 200 seeds.

**Figure 3:**
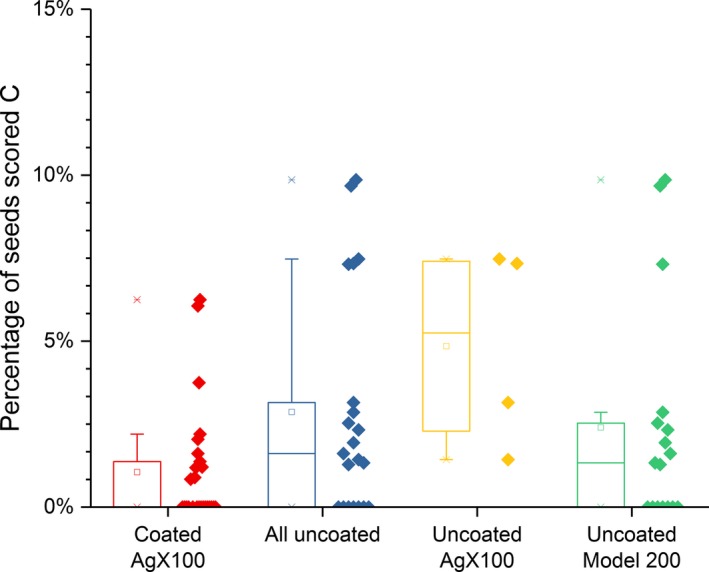
Percentages of seeds scored C for each implant, for implants using coated seeds, uncoated seeds of both nuclides, uncoated AgX100 seeds, and uncoated Model 200 seeds.

**Figure 4:**
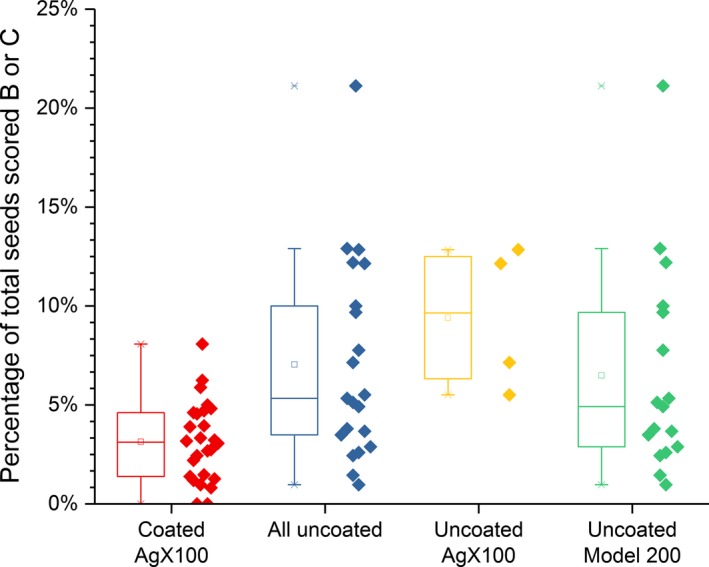
Percentages of total seeds scored either B or C for each implant, for implants using coated seeds, uncoated seeds of both nuclides, uncoated AgX100 seeds, and uncoated Model 200 seeds. Note that the range of the vertical axis used in this figure is greater than the one used in Figures 2 and 3.

Table 3 presents the comparison of the seed slippage incidence as defined above between implants with coated and all done with uncoated seeds. Table 4 presents the results of such a comparison between coated and uncoated AgX100 seeds only, while Table 5 does so between coated AgX100 seeds and (uncoated) Model 200 seeds only. Figures 2, 3, 4 present box‐and‐whisker plots for rates of relocated seeds that fulfilled the criteria for grades B, C, and the total for either B or C, respectively.

**Table 3:** Comparison between the seed slippage incidences of the coated group and uncoated group. The results for seed relocation rates categorized by B, C, and either B or C were all statistically significant, *P* < 0.05 (*P*‐values below the threshold of significance are highlighted in bold)

Group	Total # of B	Total # of C	Total # of B+C	Total seeds assessed	% of B	% of C	% of B+C
Coated AgX100 (*n* = 26)	45	25	70	2227	2.02%	1.12%	3.14%
Uncoated seeds, both models (*n* = 21)	74	54	128	1858	3.98%	2.91%	6.89%
% Difference, result of t‐test					−49.3%, ***P*** ** = 0.0026**	−61.4%, ***P*** ** = 0.033**	−54.4%, ***P*** ** = 0.0026**

**Table 4:** Comparison between the seed slippage incidences of the coated group and the subgroup of uncoated AgX100 seeds only. The results for seed relocation rates categorized by B and either B or C were statistically significant, *P* < 0.05, while that for C only was not (*P*‐values below the threshold of significance are highlighted in bold)

Group	Total # of B	Total # of C	Total # of B+C	Total seeds assessed	% of B	% of C	% of B+C
Coated AgX100 (*n* = 26)	45	25	70	2227	2.02%	1.12%	3.14%
Uncoated AgX100 (*n* = 4)	18	21	39	413	4.36%	5.08%	9.44%
% Difference, result of t‐test					−53.6%, ***P*** ** = 0.039**	−77.9%, *P* = 0.085	−66.7%, ***P*** ** = 0.038**

**Table 5:** Comparison between the seed slippage incidences of the coated group and the subgroup of (all uncoated) Model 200 seeds only. The results for seed relocation rates categorized by B and either B or C were statistically significant, *P* < 0.05, while that for C only was not (*P*‐values below the threshold of significance are highlighted in bold)

Group	Total # of B	Total # of C	Total # of B+C	Total seeds assessed	% of B	% of C	% of B+C
Coated AgX100 (*n* = 26)	45	25	70	2227	2.02%	1.12%	3.14%
Uncoated Model 200 (*n* = 17)	56	33	89	1445	3.88%	2.28%	6.16%
% Difference, result of t‐test					−47.9%, ***P*** ** = 0.013**	−50.8%, *P* = 0.14	−49.0%, ***P*** ** = 0.021**

## 4 DISCUSSION

PPB has been widely implemented as a treatment option for low‐ and intermediate‐risk prostate cancer and as a boost in men with high‐risk disease; however, seed movement and migration from the position of implantation may cause adverse events and should be minimized. In this paper, we compared the seed slippage incidence rate between coated and uncoated seeds through the use of a bio‐absorbable polymer coating with a standard Mick applicator and needles. The implanted seeds with the polymer coating are shown to have a lower incidence of seed slippage events than their uncoated counterparts as visualized on real‐time TRUS images. For both coated and uncoated seeds, the oncologist performing the implant noted that seed slippage was typically in the inferior direction along the needle track as the needle and obturator were retracted.

In this study, seeds classified “B” were taken to have moved 2–5 mm, while seeds scored “C” are thought to have moved at least 5 mm to have left the sagittal ultrasound field of view. The latter figure is estimated from the geometry of the ultrasound's probe sagittal linear array and the seed capsule length. A seed may leave the field of view and be classified “C” if a component of its direction of slippage is perpendicular to the image plane. If the slice thickness (elevational resolution) of the sagittal array averaged across the field of view is taken as roughly equal to the transverse plane aperture *t*, and if a seed of length *L* is oriented in the same direction as the slippage angle *θ* measured with respect to the axial direction of the beam (along the ultrasound beam axis), then the distance *d* the seed starting at the center of the image slice must slip to leave the slice is:

$$d=\frac{t/2}{\sin\theta }+\frac{L}{2}$$

The term of *L/2* arises from the condition that for a seed to leave the image slice, its entire length must be outside; if the center of the seed is at the edge of the image slice, half of it remains in the slice and it may be imaged. This equation reaches a minimum value for *θ *= 90°, that is, seed slippage perpendicular to the plane of the image. For *t *= 5.5 mm and *L *= 4.5 mm, the minimum value of *d* is 5 mm.^16^, ^17^, ^23^ Since this is the minimum distance to be classified “C,” and most seeds slipped in roughly the same direction as the needle, it is considered highly probable most seeds with that designation experienced greater distances of slippage. It should also be pointed out that the above quantitative estimates of seed slippage corresponding to scores of “B” and “C” are approximate, limited by the nature of the imaging method.

It should be noted that while the Model AgX100 ^125^I seeds and Model 200 ^103^Pd seeds have the same outer dimensions (0.8 mm diameter and 4.5 mm length), they have different end cap shapes: the Model AgX100 has convex, roughly hemispherical rounded end caps, while the Model 200 has concave, cupped end caps.^16^, ^17^ It has been suggested that the presence of the cupped ends on the Model 200 source has the effect of providing some seed fixity.^24^ Consequently, in addition to the comparison between all coated and all uncoated seeds, we also conducted a comparison of slippage propensity between coated and uncoated Model AgX100 seeds, as well as between coated AgX100 and uncoated Model 200 seeds. While this complicated the dosimetric analysis, it served the unique advantage of demonstrating that the polymer coating reduces seed slippage independent of other extrinsic factors such as the shape of the seed. The results of this study agree with those of Merrick et al., in that use of a polymer coating on ^125^I seeds is more effective at bringing about seed fixity than any such advantage the cupped ends of the Model 200 source provide.^5^ It was for this reason that an additional comparison was made between coated AgX100 seeds and uncoated Model 200 seeds in this study.

Table 2 presents the dosimetric parameters of the postoperative plans among the groups of this study. While t‐testing uncovered significant differences between V150 and V200 to the prostate among the studied groups, these are primarily due to differences in the dose distributions between ^125^I and ^103^Pd sources (mainly a result of the lower photon energy of ^103^Pd, 21 keV vs 28 keV for ^125^I). The results of t‐tests on the differences in postoperative urethra V100 between implants with coated and uncoated seeds are close to the *P* = 0.05 threshold of significance (*P* = 0.053); it is possible that further study with a somewhat larger dataset may demonstrate a significant difference in postimplant coverage of the urethra. The lack of significant results for t‐testing of the other dosimetric parameters of the postoperative plans is likely partially due to uncertainty in contouring the prostate on the postimplant CT datasets.

It is important to acknowledge the limitations of the study. Due to the retrospective nature of this study, it was not possible to control and randomize the patients to receive coated or uncoated seed implantation. Nor was it possible to ensure similar patient characteristics between study groups. Moreover, once the coated AgX100 seeds had been implemented at our institution, all subsequent patients receiving ^125^I were implanted with them, resulting in two separate time periods during which the coated and uncoated ^125^I seed implants were performed. (However, the uncoated ^103^Pd implants were performed over the same time period as the ^125^I implants.) Nevertheless, changes in implantation technique due to experience and learning curves of the radiation oncologist and medical physicist over the year during which data were collected are considered to have been minimal due to their 15–20 yr of current experience in our high‐volume institution.

As discussed above, the manufacturer‐reported spatial resolutions in the sagittal and axial directions are 0.5 and 1.0 mm, respectively. This, combined with the device's passing of the periodic spatial resolution tests described in TG‐128, provides good assurance that the sagittal image resolution was well below 2 mm in both the axial and lateral directions.

Study of the seed positions was made only with ultrasound images taken during implantation; further assessment of movement and migration of individual seeds in the days and months following PPB was not done. It is likely that further seed migration occurred well after the Day 0 postimplant CT imaging as described here. A dosimetric comparison of implants before and after swelling resolution of the prostate was likewise not performed. Thus, this study was not able to evaluate whether there was a significant difference in dosimetric quality between plans with coated and uncoated seeds after all seed motion and migration had ceased. However, since all patients received their postimplant CTs on Day 0 after having recovered from anesthesia, any relative change in edema between the coated and noncoated groups was avoided.

## 5 CONCLUSIONS

This study demonstrates that the polymer coating available on Model AgX100 ^125^I permanent implant seeds significantly reduces seed motion during prostate brachytherapy. While the slippage rates of coated and uncoated seeds are both considered by our group to be clinically acceptable, the improvement in seed fixity by the coating provides confidence that seed positions are maintained, reducing the risk of seed movement.

## CONFLICT OF INTEREST

No conflicts of interest.
